# The Mechanism of Oxidative Stress in Pulmonary Fibrosis and Research Progress

**DOI:** 10.3390/antiox15010142

**Published:** 2026-01-22

**Authors:** Duo Xu, Qian Wang, Meng Lyu, Chunyu Huang, Xianglin Yuan, Xinyi Chen, Yongbiao Huang

**Affiliations:** Department of Oncology, Tongji Hospital, Tongji Medical College, Huazhong University of Science and Technology, Wuhan 430030, China; d202482474@hust.edu.cn (D.X.); m202376506@hust.edu.cn (Q.W.); lyumeng@hust.edu.cn (M.L.); cyhuang@tjh.tjmu.edu.cn (C.H.); yuanxianglin@hust.edu.cn (X.Y.)

**Keywords:** oxidative stress, pulmonary fibrosis, reactive oxygen species, antioxidants

## Abstract

Pulmonary fibrosis (PF) is a group of chronic progressive lung diseases characterized by irreversible remodeling of lung tissue structure, abnormal proliferation of fibroblasts, and excessive deposition of extracellular matrix (ECM), among which idiopathic pulmonary fibrosis (IPF) is the most typical subtype. Currently, the only two clinically approved therapeutic drugs (nintedanib and pirfenidone) can only partially slow disease progression without reversing fibrotic lesions, and are associated with varying degrees of adverse effects. Oxidative stress, defined as a pathological imbalance between systemic oxidant and antioxidant systems, has been substantiated by extensive research as a pivotal mechanism driving the pathogenesis and progression of pulmonary fibrosis. This review summarizes the regulatory mechanisms of oxidative stress in pulmonary fibrosis, with a focus on its critical role in inducing and promoting fibrosis through relevant target cells and signaling pathways. We also specifically highlight the latest progress and challenges in therapeutic strategies targeting oxidative stress, and discuss next-generation therapies, including the modulation of endogenous antioxidant pathways, supplementation of exogenous antioxidants, as well as nanomaterials, exosomes, and combination therapies. We hope this review will deepen the understanding of oxidative stress and pulmonary fibrosis, and provide new directions for improving the clinical efficacy of oxidative stress-targeted therapies.

## 1. Introduction

Pulmonary fibrosis (PF) is fundamentally defined as a dysregulation of the lung tissue repair response to chronic injury, wherein the homeostatic balance of the reparative process is perturbed. Its pathogenesis is closely linked to aging and is influenced by multiple risk factors, such as genetics, smoking, occupational exposure (e.g., dust, chemical toxins), and radiation exposure [1]. At present, common types of PF include radiation-induced pulmonary fibrosis (RIPF), idiopathic pulmonary fibrosis (IPF), and silicosis. IPF is by definition “idiopathic” with rapid disease progression. Multiple factors have been reported to increase disease risk, aging being the most prominent one. Many of the predisposing factors appear to act also as triggers for acute exacerbations of the disease, which herald a poor prognosis. The 5-year survival rate of patients after diagnosis is approximately 20%, indicating an extremely poor prognosis [2]. RIPF has an incidence rate as high as 50%, which severely limits the clinical application of radiotherapy in the treatment of thoracic tumors [3]. Preventing RIPF is crucial for controlling tumor growth and improving quality of life [4]. Although silicosis has a well-defined etiology and is preventable and controllable, its pathogenic process is insidious and protracted, rendering early diagnosis and effective treatment difficult [5]. As a result, the disease is often not identified until it progresses to the fibrotic stage [6]. All types of PF share an identical disease progression trajectory, which is characterized by an initial phase of pulmonary inflammation followed by the gradual development of fibrosis [7]. Due to the complex pathogenesis of PF and the lack of effective therapeutic targets, clinical strategies for managing PF remain inadequate [8].

**Figure 1:**
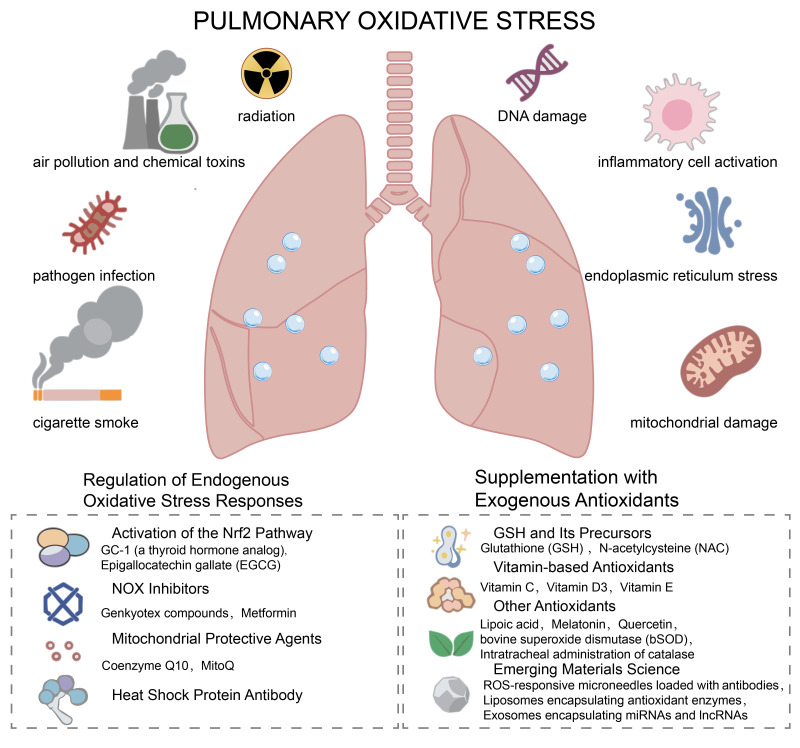
Schematic overview of pulmonary oxidative stress: inducers and therapeutic strategies targeting redox imbalance. This diagram illustrates the key inducers of pulmonary oxidative stress (left and right upper panels), including exogenous stimuli (air pollution, chemical toxins, pathogen infection, cigarette smoke, radiation) and endogenous stressors (DNA damage, inflammatory cell activation, endoplasmic reticulum stress, mitochondrial damage). Interventions targeting intrinsic antioxidant pathways, including Nrf2 pathway activators such as GC-1 (a thyroid hormone analog) and EGGG (Epigallocatechin gallate), NOX inhibitors (Genkyotex compounds, Metformin), mitochondrial protective agents (Coenzyme Q10, MitoQ), and heat shock protein antibodies. Exogenous antioxidant interventions, including glutathione (GSH) and its precursors (N-acetylcysteine, NAC), vitamin-based antioxidants (vitamin C, D3, E), other antioxidants (lipoic acid, melatonin, etc.), and emerging materials science-based approaches (ROS-responsive microneedles, antioxidant-loaded liposomes, etc.).

As a respiratory organ in direct contact with the external environment, the lung is highly susceptible to exogenous oxidative stressors (e.g., cigarette smoke, air pollution, radiation, pathogen infection, chemical toxins). Meanwhile, endogenous factors (e.g., inflammatory cell activation, mitochondrial damage, and endoplasmic reticulum stress) can also promote the production of free radicals [9]. As shown in Figure 1, under normal physiological conditions, the lungs initiate repair programs in response to stress following injury, and the proliferation of fibroblasts and synthesis of extracellular matrix (ECM) are tightly regulated to maintain the integrity of lung tissue structure. Nonetheless, when the level of oxidative stress in the lungs exceeds the compensatory capacity of the antioxidant system, it can manifest as an oxidative stress-related accelerated aging phenotype, including DNA damage, epigenetic changes, cellular senescence, protein homeostasis disruption, and mitochondrial dysfunction [10]. Free radicals directly induce apoptosis of alveolar epithelial cells, thereby initiating tissue repair cascades. Additionally, they drive the transdifferentiation of fibroblasts into myofibroblasts, accelerate excessive extracellular matrix (ECM) deposition, modulate macrophage polarization, and sustain a pro-fibrotic inflammatory microenvironment that perpetuates pathological fibrogenesis. Consequently, normal lung tissue is gradually replaced by fibrotic tissue, culminating in the progressive loss of pulmonary function, characterized by impaired gas exchange and dyspnea [11,12].

## 2. Overview of Pulmonary Oxidative Stress and Antioxidant Systems

The dynamic regulation of redox homeostasis is crucial for maintaining the normal functions of the body, and its dysregulation is a pivotal pathogenic driver of various major diseases [13,14]. Generally, low to moderate concentrations of reactive oxygen species (ROS) are beneficial for maintaining intracellular physiological activities and signaling pathways, while excessive ROS accumulation may induce malignant transformation, cellular damage, or even death. Fundamentally, oxidative stress is characterized by an imbalance between the generation and elimination of free radicals. Free radicals are defined as molecules or ions containing one or more unpaired electrons and are primarily categorized into ROS and reactive nitrogen species (RNS) [15].

ROS include both free radicals, such as the superoxide anion (O_2_^•^^−^) and the hydroxyl radical (HO^•^), and non-radical species, including hydrogen peroxide (H_2_O_2_) [16]. ROS production in cells has multiple sources. The mitochondrial ETC is the primary source of intracellular ROS [17]. Additionally, ROS are generated within the endoplasmic reticulum (ER) during physiological protein folding and ER stress [18]. In immune cells, the activation of NADPH oxidase (NOX) and dual oxidase (DUOX) enzymes during the oxidative burst leads to ROS production [19], while peroxisomes generate H_2_O_2_ and participate in substrate oxidation [20,21].

Reactive nitrogen species (RNS), a family of nitrogen-derived reactive intermediates encompassing NO^•^, NO_2_, N_2_O_3_, and ONOO^−^ [22], are mainly produced by subtypes of nitric oxide synthase (NOS), including neuronal Noxidative stress (nNOS), inducible NOS (iNOS), and endothelial NOS (eNOS). The synthesis of NO^•^ depends on molecular oxygen, L-arginine, and the cofactor tetrahydrobiopterin (BH_4_) [23]. Under conditions of L-arginine deficiency, NOS undergoes uncoupling and produces O_2_^•−^, which then combines with NO^•^ to form the highly oxidizing ONOO^−^ [24].

In the pulmonary environment, various cells are involved in the production of ROS/RNS. For example, immune cells such as macrophages and neutrophils generate reactive substances through NOX2 activation during oxidative burst to defend against pathogens [25]. Ciliated bronchial epithelial cells (BECs) express DUOX1/2 [26], whereas type 2 alveolar epithelial cells (AEC2s) can upregulate the expression of DUOX2 and NOX1/4 under pathological conditions [27]. Fibroblasts mainly produce H_2_O_2_ through NADPH oxidase 4 (NOX4) [28]. Additionally, pulmonary endothelial cells and vascular smooth muscle cells generate ROS through NOX1/2/4/5, xanthine oxidase (XO), and mitochondrial pathways, and endothelial cells also produce NO^•^ through eNOS [29,30].

Lung tissue possesses a robust detoxification mechanism to maintain oxidative balance, which is mainly divided into non-specific (non-enzymatic) and specific (enzymatic) systems. The non-enzymatic mechanism relies on small-molecule antioxidants such as glutathione (GSH), which can directly scavenge ROS and act as a coenzyme in enzymatic reactions to participate in the repair of oxidative damage [31,32]. The enzymatic mechanism consists of specialized enzyme systems, covering multiple functional families, including GSH metabolism-related enzymes, superoxide anion scavenging enzymes, hydrogen peroxide-degrading enzymes, peroxiredoxins, thioredoxins, and heme oxygenases [33]. In patients with PF, the function of this antioxidant system is significantly impaired, characterized by decreased GSH levels, reduced antioxidant enzyme activity, and insufficient activation of the nuclear factor erythroid 2-related factor 2 (Nrf2) pathway [34]. Meanwhile, the status of oxidative stress in the lungs is also associated with systemic inflammation, which is particularly prominent in patients with chronic obstructive pulmonary disease (COPD) [35]. Airway inflammation, such as the excessive production of nitric oxide in the peripheral airways, may be linked to the involvement of the distal lungs in pulmonary fibrosis [36]. In addition, pulmonary rehabilitation can reduce the risk factors for cardiovascular diseases and the incidence of cardiovascular diseases themselves, with patients experiencing acute exacerbations deriving greater benefits from pulmonary rehabilitation [37].

## 3. Key Mechanisms by Which Oxidative Stress Drives Pulmonary Fibrosis

### 3.1. Direct Injury to Pulmonary Tissue Cells and Initiation of the Fibrotic Process

Alveolar epithelial cells (AECs) are the primary target cells of oxidative stress-induced damage in the lungs, and their sustained injury serves as the initiating signal for pulmonary fibrosis [38]. ROS/RNS produced by exogenous (e.g., radiotherapy, smoking, air pollution) or endogenous (e.g., macrophage activation, mitochondrial dysfunction) sources can damage alveolar epithelial cells through multiple pathways [39]. ROS/RNS can attack membrane lipids, triggering lipid peroxidation and disrupting membrane integrity; they can also oxidize intracellular proteins, leading to loss of enzyme activity and denaturation of structural proteins. Additionally, they can damage DNA, causing gene mutations or breaks and interfering with the balance between normal cell proliferation and apoptosis. Furthermore, they can disrupt mitochondrial structure (e.g., inducing mitochondrial DNA mutations and ETC dysfunction), thereby further exacerbating ROS production [40]. These damages ultimately result in apoptosis or senescence of alveolar epithelial cells. Apoptosis of epithelial cells hinders lung tissue re-epithelialization, while senescent epithelial cells secrete senescence-associated secretory phenotype (SASP), which contains pro-fibrotic cytokines and chemokines, further activating fibroblasts and initiating the pathological process of fibrosis [41,42].

In addition, oxidative stress directly affects ECM metabolism homeostasis: ROS/RNS can induce oxidative damage to collagen and fibronectin in the ECM, thereby triggering their fragmentation or abnormal cross-linking and ultimately disrupting the normal structure and function integrity of the ECM [43]. Fragmented ECM can act as a damage signal to stimulate fibroblast activation [44,45]. Meanwhile, oxidative stress inhibits the activity of matrix metalloproteinases (MMPs, key enzymes for ECM degradation) and promotes the expression of tissue inhibitors of metalloproteinases (TIMPs, which inhibit MMP function), resulting in reduced ECM degradation and increased deposition, thereby exacerbating fibrotic remodeling of lung tissue [46].

### 3.2. Modulation of Cellular Phenotypes and Functions to Accelerate Fibrosis Progression

**Figure 2:**
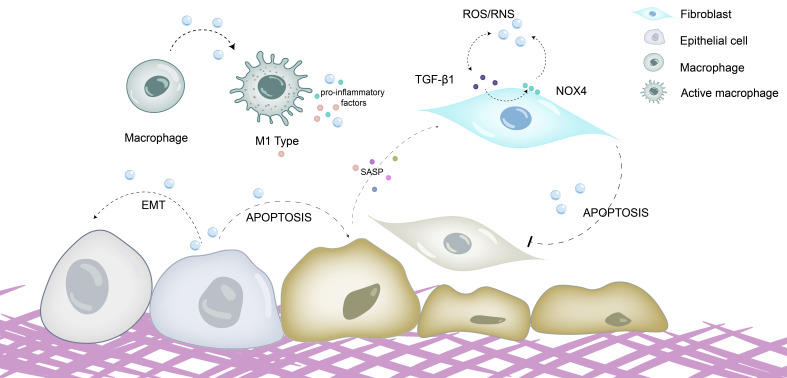
Oxidative stress promotes fibrosis by directly damaging cells and regulating cellular phenotypes and functions. ROS/RNS directly damage alveolar epithelial cells, inducing epithelial–mesenchymal transition (EMT), promoting epithelial cell senescence and apoptosis, stimulating the secretion of senescence-associated secretory phenotype (SASP), and activating fibroblasts. Meanwhile, oxidative stress can drive the polarization of macrophages toward the M1 phenotype; M1 macrophages exacerbate oxidative stress responses by generating large amounts of ROS and releasing proinflammatory cytokines such as TNF-α and IL-6. ROS/RNS can also facilitate the differentiation, senescence and apoptosis resistance of myofibroblasts. ROS is able to activate the TGF-β1/Smad pathway, thereby promoting myofibroblast formation. TGF-β1 can upregulate NOX4 expression to induce ROS production, while ROS in turn activate latent TGF-β1, forming a TGF-β1-ROS positive feedback loop that further accelerates myofibroblast activation and extracellular matrix (ECM) deposition.

As shown in Figure 2, oxidative stress plays a key regulatory role in the phenotype and function of pulmonary fibrosis-related cells, directly promoting the fibrotic process.

Alveolar epithelial cells: In addition to direct damage leading to apoptosis, oxidative stress can induce epithelial–mesenchymal transition (EMT) in alveolar epithelial cells. ROS activate signaling pathways such as Smad and PI3K/Akt, promoting epithelial cells to express mesenchymal cell markers (e.g., α-smooth muscle actin, vimentin), lose epithelial cell characteristics, and transform into cells with fibroblast functions, thereby further increasing ECM sources [47]. Meanwhile, oxidative stress synergizes with endoplasmic reticulum stress and mitochondrial dysfunction to exacerbate the senescence of alveolar epithelial cells, which continuously release pro-fibrotic factors (e.g., transforming growth factor β1 (TGF-β1), PDGF) through SASP to activate fibroblasts [48].

Fibroblasts and myofibroblasts: The transdifferentiation, senescence, and apoptosis resistance of myofibroblasts represent core markers of fibrotic progression, and oxidative stress exerts a pivotal regulatory role in orchestrating these pathological cellular processes. On the one hand, ROS, especially NOX4-mediated H_2_O_2_, are important inductive signals for the differentiation of fibroblasts into myofibroblasts [49]. ROS can activate the TGF-β1/Smad pathway, upregulate the expression of genes such as α-SMA and collagen, and promote myofibroblast formation. On the other hand, oxidative stress induces senescence but inhibits apoptosis in myofibroblasts [50]. ROS induce cellular senescence by damaging DNA and simultaneously activate anti-apoptotic pathways such as PI3K/Akt, enabling senescent myofibroblasts to survive continuously and secrete ECM, leading to long-term maintenance of fibrosis [51]. In addition, a bidirectional regulatory crosstalk exists between TGF-β1 (the core pro-fibrotic cytokine) and oxidative stress: TGF-β1 can promote ROS production by upregulating NOX4 expression, while ROS can activate latent TGF-β1 (e.g., by degrading the latency-associated peptide and activating integrins), forming a “TGF-β1-ROS” positive feedback loop that further accelerates myofibroblast activation and ECM deposition [52].

Macrophages: Alveolar macrophages (AMs) are involved in the development of IPF from the initial stage due to their direct exposure to air and response to external oxidative damage. Macrophages produce high levels of ROS under oxidative stress, which further promotes the recruitment of pulmonary macrophages [53]. Studies have shown that the lungs of IPF patients are mainly infiltrated by pro-fibrotic M2-like macrophages. Excessive oxidants not only directly damage lung cells but also induce the secretion of various chemokines and pro-fibrotic cytokines, such as chemokine ligand 2 (CCL2) and TGF-β1, and regulate macrophage polarization, thereby promoting the development of IPF. In the initial stage of tissue damage, oxidative stress (e.g., LPS- or TNF-α-induced ROS) can promote the polarization of macrophages to the M1 type (pro-inflammatory phenotype). M1 macrophages clear pathogens and initiate inflammatory responses by producing large amounts of ROS and releasing pro-inflammatory factors such as TNF-α and IL-6 [54]. In the repair stage, changes in ROS levels (e.g., H_2_O_2_ accumulation) and pro-fibrotic factors (e.g., TGF-β1, IL-4, IL-13) can induce the polarization of macrophages to the M2 type (pro-fibrotic phenotype) [55]. M2 macrophages secrete pro-fibrotic factors such as TGF-β1, PDGF, and CCL-18 to stimulate fibroblast proliferation and differentiation; they also produce TIMPs to inhibit ECM degradation. The chemokines, such as CCL2, secreted by M2 macrophages can further recruit fibroblasts and pro-fibrotic macrophages, amplifying the fibrotic response [56,57].

### 3.3. Activation of Pro-Fibrotic Signaling Pathways and the Immune Microenvironment to Sustain the Fibrotic State

**Figure 3:**
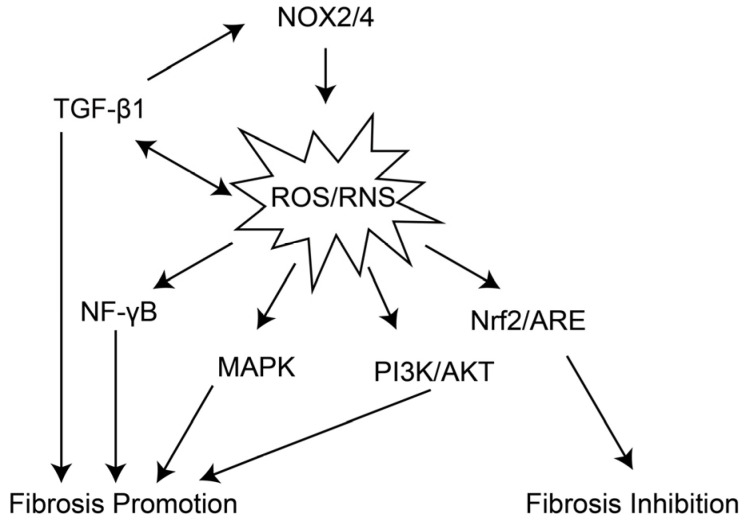
The related pathways activated by oxidative stress during the progression of pulmonary fibrosis. NOX2/4 can activate the production of ROS/RNS, while ROS/RNS and TGF-β1 form a positive feedback loop to further enhance their levels. ROS/RNS not only promote the progression of fibrosis by activating pathways such as NF-κB, MAPK, and PI3K/AKT, working in concert with TGF-β1, but also induce the Nrf2/ARE pathway to exert a fibrosis-inhibiting effect in response to their presence.

Oxidative stress serves as a key factor governing pulmonary inflammatory responses and immune homeostasis in the lung microenvironment. On the one hand, oxidative stress activates neutrophils, promoting their infiltration into lung tissue and the release of ROS and elastase, thereby exacerbating lung tissue injury [58,59]. Oxidative stress also activates the NLRP3 inflammasome, inducing pyroptosis in macrophages and alveolar epithelial cells. The pro-inflammatory factors (e.g., IL-1β, IL-18) released by pyroptotic cells can further exacerbate the inflammatory response and recruit more inflammatory cells, forming a chronic inflammatory loop that provides conditions for the continuous development of fibrosis [60]. On the other hand, oxidative stress exerts a sustained impact on the immune microenvironment. Oxidative stress promotes the differentiation of Th2 cells, which secrete pro-fibrotic cytokines such as IL-4 and IL-13, further inducing M2 macrophage polarization [61]. Concurrently, it also inhibits the function of Treg cells (regulatory T cells), weakening their anti-inflammatory effects, leading to uncontrolled inflammatory responses and shaping a pro-fibrotic immune microenvironment that supports fibrosis [62]. As shown in Figure 3, oxidative stress can activate multiple pro-fibrotic signaling pathways, regulate the expression of pulmonary fibrosis-related molecules, and form a complex molecular network [63].

TGF-β1/Smad pathway: TGF-β1 is currently recognized as the core pro-fibrotic cytokine, and OS can activate this pathway through multiple mechanisms. ROS can directly oxidize the LAP in the latent TGF-β1 complex, releasing active TGF-β1 [64]; ROS can also activate integrins (e.g., αvβ6) to mediate the activation of latent TGF-β1 [65]. Furthermore, by activating pathways such as MAPK and PI3K/Akt, ROS enhance Smad2/3 phosphorylation, promote their nuclear translocation, and upregulate the expression of downstream pro-fibrotic target genes [66]. Additionally, TGF-β1 can promote ROS production by upregulating NOX4 expression, forming a “TGF-β1-ROS-TGF-β1” positive feedback loop that continuously drives fibrosis [52].

NOX4/ROS Pathway: NOX4 is a critical ROS-generating enzyme in pulmonary fibrosis, predominantly expressed in fibroblasts, alveolar epithelial cells, and endothelial cells [67]. NOX4 expression is significantly elevated in the lung tissues of patients with IPF. The H_2_O_2_ produced by NOX4 induces the differentiation of fibroblasts into myofibroblasts and confers resistance to apoptosis, thereby inhibiting fibroblast apoptosis [68]. Concurrently, it promotes the oxidative cross-linking of ECM proteins, enhancing ECM stability, and activates the NLRP3 inflammasome, exacerbating the inflammatory response [69].

Nrf2/ARE pathway: Nrf2 is a key transcription factor for the body to resist oxidative stress. Under normal conditions, Nrf2 binds to Keap1 in the cytoplasm and remains inactive [70]. Under oxidative stress, Keap1 undergoes oxidative modification, releasing Nrf2. Nrf2 enters the nucleus and binds to ARE, activating the expression of downstream antioxidant genes and anti-fibrotic genes [71,72]. In IPF patients, the Nrf2 pathway is insufficiently activated, characterized by reduced nuclear localization of Nrf2 and decreased expression of target genes, leading to a decline in the body’s antioxidant capacity and failure to inhibit the expression of pro-fibrotic molecules such as TGF-β1 and NOX4, thereby exacerbating oxidative stress and fibrosis [73].

Other Signaling Pathways: Oxidative stress can also activate the MAPK, PI3K, and NF-κB pathways [74]. Specifically, the MAPK pathway is involved in the regulation of fibroblast proliferation and differentiation, while the NF-κB pathway maintains a pro-fibrotic inflammatory microenvironment by promoting the expression of pro-inflammatory cytokines (e.g., TNF-α, IL-6) [75]. Furthermore, oxidative stress influences cellular senescence and metabolism by modulating SIRT family proteins (e.g., SIRT1, SIRT3) [76]. Downregulation of SIRT1 exacerbates alveolar epithelial cell senescence, whereas SIRT3 deficiency results in mitochondrial dysfunction, further augmenting ROS production [77,78].

## 4. Intervention Strategies for Pulmonary Fibrosis Targeting Oxidative Stress

Current intervention strategies for PF targeting oxidative stress mainly include regulating the endogenous oxidative stress response and supplementing exogenous antioxidants, which rebalance the oxidative stress system through targeted interventions. Table 1 summarizes the clinical and preclinical evidence for pulmonary fibrosis therapies targeting oxidative stress.

### 4.1. Regulation of Endogenous Oxidative Stress Responses

The modulation of endogenous antioxidant pathways, along with the development of specific inhibitors targeting the primary sources of ROS to reduce their generation, constitutes a precision strategy for targeting oxidative stress [107].

#### 4.1.1. Activation of the Nrf2 Pathway

Nrf2 is a transcription factor recognized as the primary regulator of the cellular antioxidant responses. Augmenting the endogenous antioxidant capacity of the organism via activation of the Nrf2 signaling pathway constitutes a core therapeutic strategy for the targeted modulation of oxidative stress. In a bleomycin-induced PF mouse model, Nrf2-knockout mice exhibited more severe fibrosis [108], whereas Nrf2 activators significantly increased the activities of superoxide dismutase (SOD) and GSH peroxidase (GPx) as well as the level of GSH (GSH) in lung tissues, eliminated excessive ROS, and alleviated fibrosis [64]. Currently, multiple approaches achieve antifibrotic effects by activating Nrf2: initially, inhibiting the Keap1-Nrf2 interaction to promote Nrf2 release and nuclear translocation [109,110,111]; secondly, directly phosphorylating Nrf2 to enhance its stability and transcriptional activity [112,113]; and finally, regulating the expression of Nrf2 downstream target genes to mitigate PF [114,115]. Among these, GC-1 (a thyroid hormone analog) exerts its effects by activating Nrf2, scavenging excessive ROS, inhibiting macrophage inflammasome assembly and pyroptosis, restoring mitochondrial function in epithelial cells, thereby alleviating acute lung injury (ALI) and subsequent fibrotic progression [80]. Epigallocatechin gallate (EGCG), a polyphenol and the major bioactive component of green tea, possesses potent free radical scavenging activity against peroxides, hydroxyl radicals, peroxyl radicals, nitric oxide, carbon-centered ROS, and lipid oxidation products. It primarily exerts antioxidant, anti-inflammatory, and antifibrotic protection in various IPF models by activating the Nrf2 pathway [79,116].

#### 4.1.2. NOX Inhibitors

As is a key enzyme responsible for ROS production in pulmonary fibrosis, NOX4 induces mitochondrial ROS generation, regulates mitochondrial biogenesis, promotes anti-apoptosis, and mediates fibrotic progression [117]. NOX4 inhibitors can significantly reduce ROS levels in fibroblasts and ROS production in immune cells of IPF patients, inhibit myofibroblast differentiation and ECM synthesis, and attenuate inflammatory responses [118], while also demonstrating antifibrotic effects in animal models [81]. Simultaneously, genkyotex compounds, which act as dual inhibitors of NOX4/NOX1, have been reported to exert protective effects in various preclinical in vitro and in vivo studies [82]. Metformin possesses antioxidant potential, as it can inhibit transforming growth factor-β1 (TGF-β1)-induced NOX4 expression, ROS generation, and myofibroblast differentiation in lung fibroblasts in vitro, and mitigate bleomycin-induced pulmonary fibrosis [83]. However, Metformin has no effect on clinically relevant outcomes in patients with IPF [106].

#### 4.1.3. Mitochondrial Protective Agents

Mitochondria serve as both a major source of ROS and a target organelle for oxidative stress [119]. Mitochondrial protective agents can reduce ROS production by enhancing mitochondrial antioxidant capacity and improving electron transport chain (ETC) function. Coenzyme Q10 promotes mitochondrial ETC function, thereby reducing ROS generation. In mouse models of pulmonary fibrosis, MitoQ, a mitochondria-targeted coenzyme Q10 derivative, reduced mitochondrial ROS levels, inhibited fibroblast activation, and alleviated PF [84].

#### 4.1.4. Heat Shock Proteins Antibody

Heat shock proteins (HSPs), a conserved family of molecular chaperones encompassing HSP70 and HSP90, play crucial roles in protein folding and antioxidant defense [120]. These proteins contribute to cellular protection by preventing protein misfolding and acting as molecular chaperones under oxidative stress conditions [121]. HSP70 is widely expressed in human primary lung fibroblasts [122]. HSP90 is involved in ECM remodeling, myofibroblast differentiation, and apoptosis, and has been identified as a key molecule in the pathogenesis of PF [123]. Specific inhibition of HSP90 can suppress myofibroblast differentiation and survival, and improve PF in mice [124,125].

Interestingly, activating oxidative stress in specific cells can also be utilized for the treatment of PF. Selenite can upregulate the expression of GSH reductase and thioredoxin reductase (TrxR) in mouse lung fibroblasts, induce ROS production and apoptosis in these cells, thereby exerting a therapeutic effect on bleomycin-induced IPF [126].

### 4.2. Supplementation with Exogenous Antioxidants

The direct scavenging of ROS or the enhancement of antioxidant system function through the supplementation of exogenous antioxidant substances represents an early clinical intervention approach.

#### 4.2.1. GSH and Its Precursors

GSH (GSH) is an intracellular polypeptide with diverse functions, including detoxification, antioxidant defense, maintenance of thiol status, and regulation of cell proliferation [127]. Direct inhalation of GSH by patients can improve lung function, while oral administration can reduce oxidative stress levels [128]. N-acetylcysteine (NAC) possesses strong reducing capacity; it not only serves as a GSH precursor that can be converted to GSH in vivo to increase lung tissue GSH levels but also has direct ROS-scavenging ability and can induce Nrf2 expression [129]. Preclinical studies have shown that the combination of NAC and PFD in the treatment can improve lung function, alleviate oxidative stress-induced damage, and reduce PF [85,130]. However, other studies have demonstrated that the combination of NAC and PFD is associated with a higher incidence of photosensitivity and a faster rate of disease progression compared with pirfenidone monotherapy. The therapeutic response to NAC may vary according to the *TOLLIP* genotype [131]. The *TOLLIP* gene plays a crucial role in pulmonary host defense, which is an immune process modulated by oxidative signals. In particular, studies have found that NAC may exert therapeutic efficacy in IPF patients with the *TOLLIP* TT genotype, but it is associated with a detrimental trend in those with the CC genotype. Overall, the therapeutic potential of NAC in IPF patients remains unclear, and future pharmacogenomic trials are warranted.

#### 4.2.2. Vitamin-Based Antioxidants

Vitamin C can directly scavenge ROS [87], and vitamin E can inhibit lipid peroxidation [89]; both can alleviate PF in animal models. In addition to participating in calcium and phosphorus metabolism, vitamin D3 exerts dual effects of antifibrosis and regulation of oxidative stress, which can significantly increase lung tissue SOD activity and reduce the severity of PF [132]. However, although numerous in vitro and in vivo studies have demonstrated the benefits of using such antioxidants in the treatment of diseases such as IPF, the clinical application of antioxidants has proven mostly ineffective. Antioxidant compounds require extremely high doses to achieve physiological effects. For instance, vitamin C can be administered orally, but when the dose exceeds 500 milligrams (approximately equivalent to five oranges), its absorption rate becomes negligible, and the plasma concentration reaches a steady state of 80 micromoles. Rapid depletion of a non-regenerating non-enzymatic antioxidant, with accumulation of the oxidized form. When the store of the non-enzymatic antioxidant is exhausted, there is rapid oxidation of the cellular proteins. Therefore, in practical applications, it is difficult to achieve the desired antioxidant effects relying solely on vitamins [133].

#### 4.2.3. Other Antioxidants

Lipoic acid is capable of scavenging ROS and regenerating other antioxidants [134]. Alamandine exerts antioxidant effects by inhibiting the production of reactive oxygen species (ROS), thereby maintaining the pulmonary epithelial tissue in a state close to normal physiological conditions and alleviating pulmonary fibrosis [91]. Forsythiaside A ameliorates bleomycin-induced pulmonary fibrosis by inhibiting oxidative stress and apoptosis [92]. Curcumin reduces redox imbalance by activating the Nrf2 signaling pathway, thereby suppressing pulmonary inflammation and fibrosis [93,135]. Melatonin (N-acetyl-5-methoxytryptamine), a potent antioxidant with both lipophilic and hydrophilic properties, exerts antioxidant effects by directly scavenging ROS and RNS or indirectly upregulating the expression and activity of endogenous antioxidants [104]. Beyond supplementing naturally occurring antioxidants, synthetic compounds play a significant role. Naltrexone (NTX) is an opioid receptor antagonist. At higher doses, NTX can significantly downregulate the expression of pro-inflammatory cytokines, oxidative stress markers, and fibrotic markers, and restore the function of the body’s antioxidant defense system by increasing the levels of GSH and total antioxidant capacity [95]. The antioxidant and anti-fibrotic potential of PFD have been extensively verified in in vitro and in vivo IPF models [136]. However, these effects likely stem largely from its primary anti-fibrotic mechanisms; thus, PFD cannot be classified solely as an antioxidant drug. Quercetin is a potent direct ROS scavenger that also functions indirectly to alleviate PF by activating the Nrf2 pathway and inducing Nrf2-regulated genes [137]. Intratracheal administration of catalase in asbestos-treated mice has been proven to prevent PF by inhibiting H_2_O_2_ production by Rac1-activated inflammatory cells [96]. Subcutaneous injection of bovine superoxide dismutase (bSOD) has also been confirmed to attenuate RIPF [97].

#### 4.2.4. Emerging Materials Science

With the advancement of materials science, significant progress has been achieved in the development of ROS-based nanotherapeutics for the targeted intervention of fibrotic diseases [107,138,139]. Due to the unique composition of nanomaterials, they often carry multiple drugs to achieve multi-targeted inhibition of PF. For example, ROS-responsive lipid-polymer hybrid nanoparticles loaded with metformin and macitentan can eliminate lung ROS and prevent the transformation of endothelial cells into a fibrotic phenotype [140]; ROS-responsive microneedles loaded with integrin αvβ6-blocking antibodies target PF [56]; and ROS-responsive liposomes serve as an inhaled drug delivery nanoplatform for the treatment of idiopathic PF via the Nrf2 signaling pathway [53]. Manganese-curcumin metal–organic framework (MOF) nanocarriers, modified with M2-type profibrotic macrophage-binding peptide (M2pep) on their surface, achieve targeted recognition of M2 macrophages. These nanocarriers can eliminate approximately 80% of M2 macrophages and reduce the secretion of profibrotic factors. Meanwhile, manganese ions activate SOD activity and curcumin scavenges ROS and inhibits the NF-κB pathway. Both are released via carrier degradation, accumulate in fibrotic lung tissues, subsequently mitigate inflammatory responses and oxidative stress, and thereby exert robust antifibrotic efficacy [141]. In addition, mitochondria-targeted nanoantioxidants (such as liposomes encapsulating antioxidant enzymes) are under investigation, which can precisely deliver antioxidant substances to mitochondria for efficient ROS scavenging [142].

Cofactors essential for GSH, vitamins, and antioxidant enzyme activity can be delivered to the lungs via exosomes, playing a key role in neutralizing one of the major factors of oxidative stress. Moreover, the exosome-mediated delivery pathway enhances the ability of cells to scavenge harmful free radicals [143]. Simultaneously, exosomes encapsulating microRNAs (miRNAs) and long non-coding RNAs (lncRNAs) that target genes involved in oxidative stress pathways also contribute to combating PF [144].

## 5. Future Directions

Existing studies have clearly identified oxidative stress as a crucial driving factor in the pathological progression of PF. It participates in and amplifies the entire fibrotic process through multiple mechanisms, including direct damage to lung tissue cells, regulation of cellular phenotypes and functions, activation of profibrotic signaling pathways, and remodeling of the inflammatory and immune microenvironment. Although various drugs targeting oxidative stress have yielded promising results in preclinical experiments for inhibiting pulmonary fibrosis, monotherapy with antioxidants has been deemed unsuccessful in several clinical trials. Notably, the strategy of combining antioxidant and antifibrotic agents has yielded encouraging results in preclinical animal models, yet it has failed to demonstrate efficacy in multiple clinical trials.

This discrepancy may stem from the mismatch between the idealized conditions of animal models and the inherent complexity of clinical patients. In clinical trials, the vast majority of enrolled patients are diagnosed at the moderate to advanced stages of the disease. At this point, the pathogenic role of oxidative stress has diminished, and the core of the pathological process has shifted to the aberrant activation of fibroblasts and the irreversible deposition of the ECM. Even if ROS is scavenged by antioxidant drugs at this stage, the already formed fibrotic scars cannot be reversed, making it naturally difficult to achieve satisfactory therapeutic outcomes. Furthermore, the endpoints of animal experiments typically include pathological scores of lung tissue, hydroxyproline content, and changes in lung function—all of which can directly reflect the therapeutic efficacy of the tested drugs. In contrast, the endpoints of clinical trials generally consist of patient survival rate, time to disease progression, degree of lung function improvement, and quality of life enhancement. These clinical endpoints are influenced by multiple factors such as patient age, underlying diseases, concomitant medications, and treatment compliance, thus making it difficult to directly correlate them with the intrinsic therapeutic efficacy of the drugs. In addition, the limitations of drug delivery in clinical settings—where drugs struggle to penetrate the fibrotic barrier and reach the target lesion sites—may also contribute to the suboptimal clinical outcomes observed. On the other hand, this phenomenon may be attributed to the excessive or insufficient neutralization of the toxic effects mediated by ROS.

Encouragingly, the emergence of nanomedicine has enabled targeted strategies for the treatment of PF. The specific properties of the pulmonary fibrotic microenvironment can be recognized by Oxidative stress-responsive materials, allowing for the precise delivery of therapeutic agents. Nevertheless, nanomaterials are confronted with critical issues in clinical trials, such as biosafety, large-scale manufacturing, and cost management, which means they still have a long journey ahead to achieve clinical translation.

Given the complexity of redox mechanisms across different types of PF and distinct stages of disease progression, arbitrary modulation of the redox state may be inappropriate. Therefore, the precise regulation of redox balance holds significant importance and has become a key focus in the development of next-generation redox-targeted drugs for PF treatment. Multiple studies have indicated that the specific regulation of antioxidant pathways possesses enormous potential in reducing adverse reactions and overcoming therapeutic resistance. Despite the promising prospects, a comprehensive understanding of the mechanisms underlying redox regulation in PF progression remains challenging and requires substantial experimental validation. Additionally, individual variations may lead to differences in drug responses, making the development of personalized precision medicine an indispensable prerequisite for advancing redox medicine.

## Figures and Tables

**Table 1:** Clinical and preclinical evidence for oxidative stress-targeted therapies.

Study Type	Intervention	Sample Size	Outcomes and Safety Data	Ref.
preclinical	EGCG		Effective in animal models	[79]
preclinical	GC-1		Effective in animal models	[80]
preclinical	NOX Inhibitors		Effective in animal models	[81]
preclinical	Genkyotex		Effective in animal models	[82]
preclinical	Metformin		Effective in animal models	[83]
preclinical	MitoQ		Effective in animal models	[84]
preclinical	NAC		Effective in animal models	[85]
preclinical	Vitamins D		Effective in animal models	[86]
preclinical	Vitamins C		Effective in animal models	[87,88]
preclinical	Vitamins E		Effective in animal models	[89]
preclinical	Lipoic acid		Effective in animal models	[90]
preclinical	Alamandine		Effective in animal models	[91]
preclinical	Forsythiaside A		Effective in animal models	[92]
preclinical	Curcumin		Effective in animal models	[93]
preclinical	Melatonin		Effective in animal models	[94]
preclinical	Naltrexone		Effective in animal models	[95]
preclinical	Inhibiting H_2_O_2_ production		Effective in animal models	[96]
preclinical	Subcutaneous injection of bovine superoxide dismutase (bSOD)		Effective in animal models	[97]
clinical	Metformin	624	No effect on clinically relevant outcomes	[98]
clinical	GSH	105	Slight positive effects	[99]
clinical	Combination therapy with inhaled NAC and PFD	81	Worse outcomes for IPF	[100]
clinical	NAC	564	Decrease lung inflammation and fibrosis	[101]
clinical	NAC	66	No evidence of reduced indicators of inflammation or oxidative stress	[102]
clinical	Aerosolized NAC	30	Delay disease progression	[103]
clinical	Combination therapy with NAC and PFD	123	A significantly higher incidence of photosensitivity and experienced more rapid disease progression	[104]
clinical	Supplementation of vitamins D, C and E	33	Positively affect the respiratory function and alleviate the inflammation and oxidative stress	[105]
clinical	Antioxidant-enriched multivitamin	73	No significant efficacy was observed in terms of lung function and growth-related endpoints, whereas it could decrease the risk of acute pulmonary exacerbations in patients	[106]

## Data Availability

No new data were created or analyzed in this study. Data sharing is not applicable to this article.

## Abbreviations

The following abbreviations are used in this manuscript:

PF	Pulmonary fibrosis
IPF	Idiopathic pulmonary fibrosis
RIPF	Radiation-induced pulmonary fibrosis
ECM	Extracellular matrix
ROS	Reactive oxygen species
RNS	Reactive nitrogen species
O_2_^•−^	Superoxide anion
HO^•^	Hydroxyl radical
H_2_O_2_	Hydrogen peroxide
ETC	Electron transport chain
ER	Endoplasmic reticulum
NOX	NADPH oxidase
DUOX	Dual oxidase
NOS	Nitric oxide synthase
nNOS	Neuronal nitric oxide synthase
iNOS	Inducible nitric oxide synthase
eNOS	Endothelial nitric oxide synthase
BH_4_	Tetrahydrobiopterin
ONOO^−^	Peroxynitrite
BECs	Ciliated bronchial epithelial cells
AEC2s	Type 2 alveolar epithelial cells
NOX4	NADPH oxidase 4
XO	Xanthine oxidase
GSH	Glutathione
Nrf2	Nuclear factor erythroid 2-related factor 2
AECs	Alveolar epithelial cells
SASP	Senescence-associated secretory phenotype
MMPs	Matrix metalloproteinases
TIMPs	Tissue inhibitors of metalloproteinases
EMT	Epithelial–mesenchymal transition
PI3K/Akt	Phosphoinositide 3-kinase/Protein kinase B
α-SMA	α-smooth muscle actin
TGF-β1	Transforming growth factor β1
PDGF	Platelet-derived growth factor
AMs	Alveolar macrophages
CCL2	Chemokine ligand 2
LPS	Lipopolysaccharide
TNF-α	Tumor necrosis factor α
IL-6	Interleukin 6
IL-4	Interleukin 4
IL-13	Interleukin 13
CCL-18	Chemokine ligand 18
NLRP3	NOD-like receptor family pyrin domain-containing 3
IL-1β	Interleukin 1β
IL-18	Interleukin 18
Th2	T helper 2 cells
Treg	Regulatory T cells
MAPK	Mitogen-activated protein kinase
NF-κB	Nuclear factor κB
SIRT	Sirtuin
LAP	Latency-associated peptide
ARE	Antioxidant response element
Keap1	Kelch-like ECH-associated protein 1
SOD	Superoxide dismutase
GPx	GSH peroxidase
ALI	Acute lung injury
EGCG	Epigallocatechin gallate
HSPs	Heat shock proteins
HSP70	Heat shock protein 70
HSP90	Heat shock protein 90
TrxR	Thioredoxin reductase
NAC	N-acetylcysteine
PFD	Pirfenidone
MOF	Metal–organic framework
M2pep	M2-type profibrotic macrophage-binding peptide
miRNAs	MicroRNAs
lncRNAs	Long non-coding RNAs

## References

[1] Raghu G., Collard H.R., Egan J.J., Martinez F.J., Behr J., Brown K.K., Colby T.V., Cordier J.F., Flaherty K.R., Lasky J.A. (2011). An official ATS/ERS/JRS/ALAT statement: Idiopathic pulmonary fibrosis: Evidence-based guidelines for diagnosis and management. Am. J. Respir. Crit. Care Med..

[2] Moss B.J., Ryter S.W., Rosas I.O. (2022). Pathogenic Mechanisms Underlying Idiopathic Pulmonary Fibrosis. Annu. Rev. Pathol..

[3] Wang P., Yan Z., Zhou P.K., Gu Y. (2022). The Promising Therapeutic Approaches for Radiation-Induced Pulmonary Fibrosis: Targeting Radiation-Induced Mesenchymal Transition of Alveolar Type II Epithelial Cells. Int. J. Mol. Sci..

[4] Song X., Zhu H., Teng G. (2025). Current status and future prospects of interventional radiology in China. Oncol. Transl. Med..

[5] Jamshidi P., Danaei B., Arbabi M., Mohammadzadeh B., Khelghati F., Akbari Aghababa A., Nayebzade A., Shahidi Bonjar A.H., Centis R., Sotgiu G. (2025). Silicosis and tuberculosis: A systematic review and meta-analysis. Pulmonology.

[6] Li T., Yang X., Xu H., Liu H. (2022). Early Identification, Accurate Diagnosis, and Treatment of Silicosis. Can. Respir. J..

[7] Liu Y., Wang D., Liu X., Yuan H., Liu D., Hu Y., Ning S. (2024). Biological and pharmacological roles of pyroptosis in pulmonary inflammation and fibrosis: Recent advances and future directions. Cell Commun. Signal. CCS.

[8] Jarzebska N., Karetnikova E.S., Markov A.G., Kasper M., Rodionov R.N., Spieth P.M. (2020). Scarred Lung. An Update on Radiation-Induced Pulmonary Fibrosis. Front. Med..

[9] Su L., Dong Y., Wang Y., Wang Y., Guan B., Lu Y., Wu J., Wang X., Li D., Meng A. (2021). Potential role of senescent macrophages in radiation-induced pulmonary fibrosis. Cell Death Dis..

[10] Pokharel M.D., Marciano D.P., Fu P., Franco M.C., Unwalla H., Tieu K., Fineman J.R., Wang T., Black S.M. (2023). Metabolic reprogramming, oxidative stress, and pulmonary hypertension. Redox Biol..

[11] Su W., Guo Y., Wang Q., Ma L., Zhang Q., Zhang Y., Geng Y., Jin T., Guo J., Yang R. (2024). YAP1 inhibits the senescence of alveolar epithelial cells by targeting Prdx3 to alleviate pulmonary fibrosis. Exp. Mol. Med..

[12] Saha P., Talwar P. (2024). Idiopathic pulmonary fibrosis (IPF): Disease pathophysiology, targets, and potential therapeutic interventions. Mol. Cell. Biochem..

[13] Hu P., Song G., Chen B., Miao J. (2023). Development of a redox-related prognostic signature for predicting biochemical-recurrence-free survival of prostate cancer*. Oncol. Transl. Med..

[14] Zhou J., Wang B., Su R., Zhang S., Ji Y., Ju M., Gao Y. (2025). Research status of colorectal cancer treatment based on natural killer cell immunotherapy. Oncol. Transl. Med..

[15] Jomova K., Raptova R., Alomar S.Y., Alwasel S.H., Nepovimova E., Kuca K., Valko M. (2023). Reactive oxygen species, toxicity, oxidative stress, and antioxidants: Chronic diseases and aging. Arch. Toxicol..

[16] Sun Z., Ji Z., Meng H., He W., Li B., Pan X., Zhou Y., Yu G. (2024). Lactate facilitated mitochondrial fission-derived ROS to promote pulmonary fibrosis via ERK/DRP-1 signaling. J. Transl. Med..

[17] Okoye C.N., Koren S.A., Wojtovich A.P. (2023). Mitochondrial complex I ROS production and redox signaling in hypoxia. Redox Biol..

[18] Wu Q., Liu C., Liu D., Wang Y., Qi H., Liu X., Zhang Y., Chen H., Zeng Y., Li J. (2024). Polystyrene nanoplastics-induced lung apoptosis and ferroptosis via ROS-dependent endoplasmic reticulum stress. Sci. Total Environ..

[19] Sirokmány G., Donkó Á., Geiszt M. (2016). Nox/Duox Family of NADPH Oxidases: Lessons from Knockout Mouse Models. Trends Pharmacol. Sci..

[20] DiGiovanni L.F., Khroud P.K., Carmichael R.E., Schrader T.A., Gill S.K., Germain K., Jomphe R.Y., Wiesinger C., Boutry M., Kamoshita M. (2025). ROS transfer at peroxisome-mitochondria contact regulates mitochondrial redox. Science.

[21] Wang J., Wu Z., Zhu M., Zhao Y., Xie J. (2024). ROS induced pyroptosis in inflammatory disease and cancer. Front. Immunol..

[22] Khan M., Ali S., Al Azzawi T.N.I., Saqib S., Ullah F., Ayaz A., Zaman W. (2023). The Key Roles of ROS and RNS as a Signaling Molecule in Plant-Microbe Interactions. Antioxidants.

[23] Che Z., Zhou Z., Li S.Q., Gao L., Xiao J., Wong N.K. (2023). ROS/RNS as molecular signatures of chronic liver diseases. Trends Mol. Med..

[24] Kalyanaraman B., Cheng G., Hardy M. (2024). Gut microbiome, short-chain fatty acids, alpha-synuclein, neuroinflammation, and ROS/RNS: Relevance to Parkinson’s disease and therapeutic implications. Redox Biol..

[25] Li J., Li M., Zhang C., Fei Y., Wang Y., Zhong Z., Peng C., Li M., Gui S., Guo J. (2024). Active targeting microemulsion-based thermosensitive hydrogel against periodontitis by reconstructing Th17/Treg homeostasis via regulating ROS-macrophages polarization cascade. Int. J. Pharm..

[26] Sul O.J., Ra S.W. (2021). Quercetin Prevents LPS-Induced Oxidative Stress and Inflammation by Modulating NOX2/ROS/NF-kB in Lung Epithelial Cells. Molecules.

[27] Schiffers C., van de Wetering C., Bauer R.A., Habibovic A., Hristova M., Dustin C.M., Lambrichts S., Vacek P.M., Wouters E.F., Reynaert N.L. (2021). Downregulation of epithelial DUOX1 in chronic obstructive pulmonary disease. JCI Insight.

[28] Li Y., Liu C., Rolling L., Sikora V., Chen Z., Gurwin J., Barabell C., Lin J., Duan C. (2023). ROS signaling-induced mitochondrial Sgk1 expression regulates epithelial cell renewal. Proc. Natl. Acad. Sci. USA.

[29] Nijmeh J., Moldobaeva A., Wagner E.M. (2010). Role of ROS in ischemia-induced lung angiogenesis. Am. J. Physiol. Lung Cell. Mol. Physiol..

[30] Zemskov E.A., Lu Q., Ornatowski W., Klinger C.N., Desai A.A., Maltepe E., Yuan J.X., Wang T., Fineman J.R., Black S.M. (2019). Biomechanical Forces and Oxidative Stress: Implications for Pulmonary Vascular Disease. Antioxid. Redox Signal..

[31] Nagar E., Singh N., Saini N., Arora N. (2025). Glutathione attenuates diesel exhaust-induced lung epithelial injury via NF-κB/Nrf2/GPX4-mediated ferroptosis. Toxicology.

[32] Sajid S., Chen X., Sun Y., Luo J., Zhang B., Chen L., Huang J., Lai C., Chen Y., Guo L. (2025). A translational in vitro to in vivo study on chronic arsenic exposure induced pulmonary ferroptosis and multi-omics analysis of gut-lung axis correlation. J. Hazard. Mater..

[33] Sobczak M., Strachowska M., Gronkowska K., Karwaciak I., Pułaski Ł., Robaszkiewicz A. (2021). LSD1 Facilitates Pro-Inflammatory Polarization of Macrophages by Repressing Catalase. Cells.

[34] Mohanan A., Washimkar K.R., Mugale M.N. (2024). Unraveling the interplay between vital organelle stress and oxidative stress in idiopathic pulmonary fibrosis. Biochim. Et Biophys. Acta Mol. Cell Res..

[35] Austin V., Crack P.J., Bozinovski S., Miller A.A., Vlahos R. (2016). COPD and stroke: Are systemic inflammation and oxidative stress the missing links?. Clin. Sci..

[36] Hirano T., Matsunaga K., Sugiura H., Minakata Y., Koarai A., Akamatsu K., Ichikawa T., Furukawa K., Ichinose M. (2013). Relationship between alveolar nitric oxide concentration in exhaled air and small airway function in COPD. J. Breath Res..

[37] Muñoz Montiel A., Ruiz-Esteban P., Doménech Del Río A., Valdivielso P., Sánchez Chaparro M., Olveira C. (2024). The effect of pulmonary rehabilitation on cardiovascular risk, oxidative stress and systemic inflammation in patients with COPD. Respir. Med..

[38] Wang L., Yuan H., Li W., Yan P., Zhao M., Li Z., Zhao H., Wang S., Wan R., Li Y. (2024). ACSS3 regulates the metabolic homeostasis of epithelial cells and alleviates pulmonary fibrosis. Biochim. Et Biophys. Acta Mol. Basis Dis..

[39] Albrecht C., Knaapen A.M., Becker A., Höhr D., Haberzettl P., van Schooten F.J., Borm P.J., Schins R.P. (2005). The crucial role of particle surface reactivity in respirable quartz-induced reactive oxygen/nitrogen species formation and APE/Ref-1 induction in rat lung. Respir. Res..

[40] Zheng D., Liu J., Piao H., Zhu Z., Wei R., Liu K. (2022). ROS-triggered endothelial cell death mechanisms: Focus on pyroptosis, parthanatos, and ferroptosis. Front. Immunol..

[41] Cui J., Xu Z., Yu Z., Zhang Q., Liu S., Du B., Gan L., Yan C., Xue G., Feng J. (2025). High-alcohol-producing Klebsiella pneumoniae aggravates lung injury by affecting neutrophils and the airway epithelium. Cell Rep. Med..

[42] Fu P., Ramchandran R., Sudhadevi T., Kumar P.P.K., Krishnan Y., Liu Y., Zhao Y., Parinandi N.L., Harijith A., Sadoshima J. (2021). NOX4 Mediates Pseudomonas aeruginosa-Induced Nuclear Reactive Oxygen Species Generation and Chromatin Remodeling in Lung Epithelium. Antioxidants.

[43] Maher T.M., Assassi S., Azuma A., Cottin V., Hoffmann-Vold A.M., Kreuter M., Oldham J.M., Richeldi L., Valenzuela C., Wijsenbeek M.S. (2025). Nerandomilast in Patients with Progressive Pulmonary Fibrosis. N. Engl. J. Med..

[44] Tomos I., Kanellopoulou P., Nastos D., Aidinis V. (2025). Pharmacological targeting of ECM homeostasis, fibroblast activation and invasion for the treatment of pulmonary fibrosis. Expert Opin. Ther. Targets.

[45] Chen C., Yang S., Zhang M., Zhang Z., Hong J., Han D., Ma J., Zhang S.B., Okunieff P., Zhang L. (2016). Triptolide mitigates radiation-induced pulmonary fibrosis via inhibition of axis of alveolar macrophages-NOXes-ROS-myofibroblasts. Cancer Biol. Ther..

[46] Zhang H., Shen B., Swinarska J.T., Li W., Xiao K., He P. (2014). 9-Hydroxypheophorbide α-mediated photodynamic therapy induces matrix metalloproteinase-2 (MMP-2) and MMP-9 down-regulation in Hep-2 cells via ROS-mediated suppression of the ERK pathway. Photodiagn. Photodyn. Ther..

[47] Yuan R., Fan Q., Liang X., Han S., He J., Wang Q.Q., Gao H., Feng Y., Yang S. (2022). Cucurbitacin B inhibits TGF-β1-induced epithelial-mesenchymal transition (EMT) in NSCLC through regulating ROS and PI3K/Akt/mTOR pathways. Chin. Med..

[48] Mittal M., Siddiqui M.R., Tran K., Reddy S.P., Malik A.B. (2014). Reactive oxygen species in inflammation and tissue injury. Antioxid. Redox Signal..

[49] Gao F., Pan L., Liu W., Chen J., Wang Y., Li Y., Liu Y., Hua Y., Li R., Zhang T. (2025). Idiopathic pulmonary fibrosis microenvironment: Novel mechanisms and research directions. Int. Immunopharmacol..

[50] Macip S., Igarashi M., Fang L., Chen A., Pan Z.Q., Lee S.W., Aaronson S.A. (2002). Inhibition of p21-mediated ROS accumulation can rescue p21-induced senescence. EMBO J..

[51] Zhan J.H., Wei J., Liu L., Xu Y.T., Ji H., Wang C.N., Liu Y.J., Zhu X.Y. (2023). Investigation of a UPR-Related Gene Signature Identifies the Pro-Fibrotic Effects of Thrombospondin-1 by Activating CD47/ROS/Endoplasmic Reticulum Stress Pathway in Lung Fibroblasts. Antioxidants.

[52] Vermot A., Petit-Härtlein I., Smith S.M.E., Fieschi F. (2021). NADPH Oxidases (NOX): An Overview from Discovery, Molecular Mechanisms to Physiology and Pathology. Antioxidants.

[53] Liu J., Wu Z., Liu Y., Zhan Z., Yang L., Wang C., Jiang Q., Ran H., Li P., Wang Z. (2022). ROS-responsive liposomes as an inhaled drug delivery nanoplatform for idiopathic pulmonary fibrosis treatment via Nrf2 signaling. J. Nanobiotechnol..

[54] Li C., Deng C., Wang S., Dong X., Dai B., Guo W., Guo Q., Feng Y., Xu H., Song X. (2024). A novel role for the ROS-ATM-Chk2 axis mediated metabolic and cell cycle reprogramming in the M1 macrophage polarization. Redox Biol..

[55] Du S.L., Zhou Y.T., Hu H.J., Lin L., Zhang Z.Q. (2025). Silica-induced ROS in alveolar macrophages and its role on the formation of pulmonary fibrosis via polarizing macrophages into M2 phenotype: A review. Toxicol. Mech. Methods.

[56] Chen Y., Wang T., Liang F., Han J., Lou Z., Yu Y., Li J., Zhan T., Gu Y., Dong L. (2024). Nicotinamide phosphoribosyltransferase prompts bleomycin-induced pulmonary fibrosis by driving macrophage M2 polarization in mice. Theranostics.

[57] Ge Z., Chen Y., Ma L., Hu F., Xie L. (2024). Macrophage polarization and its impact on idiopathic pulmonary fibrosis. Front. Immunol..

[58] Racanelli A.C., Kikkers S.A., Choi A.M.K., Cloonan S.M. (2018). Autophagy and inflammation in chronic respiratory disease. Autophagy.

[59] Wang W., Liu Z., Zhang Y., Wang L., Meng D., Li X., Zhang J., Wu Y., Zhou X., Liu G. (2022). Benzyl butyl phthalate (BBP) induces lung injury and fibrosis through neutrophil extracellular traps. Environ. Pollut..

[60] Liu W., Han X., Li Q., Sun L., Wang J. (2022). Iguratimod ameliorates bleomycin-induced pulmonary fibrosis by inhibiting the EMT process and NLRP3 inflammasome activation. Biomed. Pharmacother. Biomed. Pharmacother..

[61] Fukuhara K., Nakashima T., Abe M., Masuda T., Hamada H., Iwamoto H., Fujitaka K., Kohno N., Hattori N. (2017). Suplatast tosilate protects the lung against hyperoxic lung injury by scavenging hydroxyl radicals. Free Radic. Biol. Med..

[62] Ding H., Xu X., Zhu Y., Ling X., Xu L. (2025). Inhibition of Alkbh5 Attenuates Lipopolysaccharide-Induced Lung Injury by Promoting Ccl1 m6A and Treg Recruitment. Cell Prolif..

[63] Zhao W., Bai B., Li H., Feng Y., Sun J., Fang Y., Zheng P., Zhang G. (2025). The role of oxidative stress-related genes in idiopathic pulmonary fibrosis. Sci. Rep..

[64] Pociask D.A., Sime P.J., Brody A.R. (2004). Asbestos-derived reactive oxygen species activate TGF-β1. Lab. Investig..

[65] Ding H., Cui Y., Yang J., Li Y., Zhang H., Ju S., Ren X., Ding C., Zhao J. (2023). ROS-responsive microneedles loaded with integrin avβ6-blocking antibodies for the treatment of pulmonary fibrosis. J. Control. Release.

[66] Xu W., Ye S., Liu W., Guo H., Zhang L., Wei S., Anwaier A., Chang K., Malafaia G., Zhang H. (2024). Single-cell RNA-seq analysis decodes the kidney microenvironment induced by polystyrene microplastics in mice receiving a high-fat diet. J. Nanobiotechnol..

[67] Dong J., Viswanathan S., Adami E., Singh B.K., Chothani S.P., Ng B., Lim W.W., Zhou J., Tripathi M., Ko N.S.J. (2021). Hepatocyte-specific IL11 cis-signaling drives lipotoxicity and underlies the transition from NAFLD to NASH. Nat. Commun..

[68] Li L., Lu M., Peng Y., Huang J., Tang X., Chen J., Li J., Hong X., He M., Fu H. (2023). Oxidatively stressed extracellular microenvironment drives fibroblast activation and kidney fibrosis. Redox Biol..

[69] Meng Y., Li T., Zhou G.S., Chen Y., Yu C.H., Pang M.X., Li W., Li Y., Zhang W.Y., Li X. (2015). The angiotensin-converting enzyme 2/angiotensin (1-7)/Mas axis protects against lung fibroblast migration and lung fibrosis by inhibiting the NOX4-derived ROS-mediated RhoA/Rho kinase pathway. Antioxid. Redox Signal..

[70] Adinolfi S., Patinen T., Jawahar Deen A., Pitkänen S., Härkönen J., Kansanen E., Küblbeck J., Levonen A.L. (2023). The KEAP1-NRF2 pathway: Targets for therapy and role in cancer. Redox Biol..

[71] Makena P., Kikalova T., Prasad G.L., Baxter S.A. (2023). Oxidative Stress and Lung Fibrosis: Towards an Adverse Outcome Pathway. Int. J. Mol. Sci..

[72] Wang X.C., Zhang Y.S., Ling H., You J.B., Cheng J., Liu Z.Y., Liu Z.Y., Lin L.C., Mao S., Liu P. (2025). Epigenetic silencing of SOD2 exacerbates mitochondrial oxidative stress and promotes pulmonary fibrosis. Free Radic. Biol. Med..

[73] Chen J., Li X., Liu H., Zhong D., Yin K., Li Y., Zhu L., Xu C., Li M., Wang C. (2023). Bone marrow stromal cell-derived exosomal circular RNA improves diabetic foot ulcer wound healing by activating the nuclear factor erythroid 2-related factor 2 pathway and inhibiting ferroptosis. Diabet. Med..

[74] Pan L., Cheng Y., Yang W., Wu X., Zhu H., Hu M., Zhang Y., Zhang M. (2023). Nintedanib Ameliorates Bleomycin-Induced Pulmonary Fibrosis, Inflammation, Apoptosis, and Oxidative Stress by Modulating PI3K/Akt/mTOR Pathway in Mice. Inflammation.

[75] Zoulikha M., Xiao Q., Boafo G.F., Sallam M.A., Chen Z., He W. (2022). Pulmonary delivery of siRNA against acute lung injury/acute respiratory distress syndrome. Acta Pharm. Sin. B.

[76] Bindu S., Pillai V.B., Kanwal A., Samant S., Mutlu G.M., Verdin E., Dulin N., Gupta M.P. (2017). SIRT3 blocks myofibroblast differentiation and pulmonary fibrosis by preventing mitochondrial DNA damage. Am. J. Physiol. Lung Cell. Mol. Physiol..

[77] Zhang Y., Li T., Pan M., Wang W., Huang W., Yuan Y., Xie Z., Chen Y., Peng J., Li X. (2022). SIRT1 prevents cigarette smoking-induced lung fibroblasts activation by regulating mitochondrial oxidative stress and lipid metabolism. J. Transl. Med..

[78] Zhang H.X., Li Y.N., Wang X.L., Ye C.L., Zhu X.Y., Li H.P., Yang T., Liu Y.J. (2019). Probucol ameliorates EMT and lung fibrosis through restoration of SIRT3 expression. Pulm. Pharmacol. Ther..

[79] Burgy O., Königshoff M. (2024). Teatime: Epigallocatechin gallate targets fibroblast-epithelial cell crosstalk to combat lung fibrosis. J. Clin. Investig..

[80] Li B., Liu J., He W., Zhou Y., Zhao M., Xia C., Pan X., Ji Z., Duan R., Lian H. (2025). Inhibition of macrophage inflammasome assembly and pyroptosis with GC-1 ameliorates acute lung injury. Theranostics.

[81] Kilic T., Parlakpinar H., Taslidere E., Yildiz S., Polat A., Vardi N., Colak C., Ermis H. (2015). Protective and therapeutic effect of apocynin on bleomycin-induced lung fibrosis in rats. Inflammation.

[82] Cui Y., Wang Y., Li G., Ma W., Zhou X.S., Wang J., Liu B. (2018). The Nox1/Nox4 inhibitor attenuates acute lung injury induced by ischemia-reperfusion in mice. PLoS ONE.

[83] Rangarajan S., Bone N.B., Zmijewska A.A., Jiang S., Park D.W., Bernard K., Locy M.L., Ravi S., Deshane J., Mannon R.B. (2018). Metformin reverses established lung fibrosis in a bleomycin model. Nat. Med..

[84] Jiang Y., Huang Z., Zhou T., Wu M., Zhao J., Xiong Z., Wang R., Chen L., Weng X., Lin L. (2025). Mitochondria-target ubiquinone attenuates bleomycin-induced pulmonary fibrosis. Front. Pharmacol..

[85] Choi S.M., Lee P.H., An M.H., Yun-Gi L., Park S., Baek A.R., Jang A.S. (2022). N-acetylcysteine decreases lung inflammation and fibrosis by modulating ROS and Nrf2 in mice model exposed to particulate matter. Immunopharmacol. Immunotoxicol..

[86] Tzilas V., Bouros E., Barbayianni I., Karampitsakos T., Kourtidou S., Ntassiou M., Ninou I., Aidinis V., Bouros D., Tzouvelekis A. (2019). Vitamin D prevents experimental lung fibrosis and predicts survival in patients with idiopathic pulmonary fibrosis. Pulm. Pharmacol. Ther..

[87] Ma L., Jin Y., Aili A., Xu L., Wang X., Xiao L., Zhao W., Yin S., Liu B., Yuan X. (2024). High-dose vitamin C attenuates radiation-induced pulmonary fibrosis by targeting S100A8 and S100A9. Biochim. Et Biophys. Acta Mol. Basis Dis..

[88] Rodrigues da Silva M., Schapochnik A., Peres Leal M., Esteves J., Bichels Hebeda C., Sandri S., Pavani C., Ratto Tempestini Horliana A.C., Farsky S.H.P., Lino-Dos-Santos-Franco A. (2018). Beneficial effects of ascorbic acid to treat lung fibrosis induced by paraquat. PLoS ONE.

[89] Chang J., Wang J., Luo B., Li W., Xiong Z., Du C., Wang X., Wang Y., Tian J., Li S. (2023). Vitamin E stabilizes iron and mitochondrial metabolism in pulmonary fibrosis. Front. Pharmacol..

[90] Zhao Y., Xu G., Li H., Chang M., Guan Y., Li Y., Wu W., Yao S. (2020). Overexpression of endogenous lipoic acid synthase attenuates pulmonary fibrosis induced by crystalline silica in mice. Toxicol. Lett..

[91] Blanco A., Fernandes R., Guimarães G.R., Rigatto K. (2025). Alamandine reduces oxidative stress and preserves the epithelium in BLM-induced pulmonary fibrosis. Eur. J. Pharmacol..

[92] Yang F., Zhang Q., Wang X., Hu Y., Chen S. (2024). Forsythiaside A ameliorates bleomycin-induced pulmonary fibrosis by inhibiting oxidative stress and apoptosis. Immun. Inflamm. Dis..

[93] Lee J.C., Kinniry P.A., Arguiri E., Serota M., Kanterakis S., Chatterjee S., Solomides C.C., Javvadi P., Koumenis C., Cengel K.A. (2010). Dietary curcumin increases antioxidant defenses in lung, ameliorates radiation-induced pulmonary fibrosis, and improves survival in mice. Radiat. Res..

[94] Yildirim Z., Kotuk M., Erdogan H., Iraz M., Yagmurca M., Kuku I., Fadillioglu E. (2006). Preventive effect of melatonin on bleomycin-induced lung fibrosis in rats. J. Pineal Res..

[95] Mohammadi Hamaneh A., Nejati F., Teymoori Masuleh M., Manavi M.A., Kazemzadeh H., Shafaroodi H., Tavangar S.M., Dehpour A.R. (2025). Naltrexone reduces bleomycin-induced lung fibrosis in rats by attenuating fibrosis, inflammation, oxidative stress, and extracellular matrix remodeling. Int. Immunopharmacol..

[96] Murthy S., Adamcakova-Dodd A., Perry S.S., Tephly L.A., Keller R.M., Metwali N., Meyerholz D.K., Wang Y., Glogauer M., Thorne P.S. (2009). Modulation of reactive oxygen species by Rac1 or catalase prevents asbestos-induced pulmonary fibrosis. Am. J. Physiol. Lung Cell. Mol. Physiol..

[97] Antonic V., Rabbani Z.N., Jackson I.L., Vujaskovic Z. (2015). Subcutaneous administration of bovine superoxide dismutase protects lungs from radiation-induced lung injury. Free Radic. Res..

[98] Spagnolo P., Kreuter M., Maher T.M., Wuyts W., Bonella F., Corte T.J., Kopf S., Weycker D., Kirchgaessler K.U., Ryerson C.J. (2018). Metformin Does Not Affect Clinically Relevant Outcomes in Patients with Idiopathic Pulmonary Fibrosis. Respir. Int. Rev. Thorac. Dis..

[99] Calabrese C., Tosco A., Abete P., Carnovale V., Basile C., Magliocca A., Quattrucci S., De Sanctis S., Alatri F., Mazzarella G. (2015). Randomized, single blind, controlled trial of inhaled glutathione vs placebo in patients with cystic fibrosis. J. Cyst. Fibros..

[100] Sakamoto S., Kataoka K., Kondoh Y., Kato M., Okamoto M., Mukae H., Bando M., Suda T., Yatera K., Tanino Y. (2021). Pirfenidone plus inhaled N-acetylcysteine for idiopathic pulmonary fibrosis: A randomised trial. Eur. Respir. J..

[101] Sun T., Liu J., Zhao W. (2016). Efficacy of N-Acetylcysteine in Idiopathic Pulmonary Fibrosis: A Systematic Review and Meta-Analysis. Medicine.

[102] Alfonso H., Franklin P., Ching S., Croft K., Burcham P., Olsen N., Reid A., Joyce D., de Klerk N., Musk A.B. (2015). Effect of N-acetylcysteine supplementation on oxidative stress status and alveolar inflammation in people exposed to asbestos: A double-blind, randomized clinical trial. Respirology.

[103] Tomioka H., Kuwata Y., Imanaka K., Hashimoto K., Ohnishi H., Tada K., Sakamoto H., Iwasaki H. (2005). A pilot study of aerosolized N-acetylcysteine for idiopathic pulmonary fibrosis. Respirology.

[104] Behr J., Bendstrup E., Crestani B., Günther A., Olschewski H., Sköld C.M., Wells A., Wuyts W., Koschel D., Kreuter M. (2016). Safety and tolerability of acetylcysteine and pirfenidone combination therapy in idiopathic pulmonary fibrosis: A randomised, double-blind, placebo-controlled, phase 2 trial. Lancet Respir. Med..

[105] Yavari M., Mousavi S.A.J., Janani L., Feizy Z., Vafa M. (2022). Effects of supplementation of vitamins D, C and E on Idiopathic Pulmonary Fibrosis (IPF): A clinical trial. Clin. Nutr. Espen.

[106] Sagel S.D., Khan U., Jain R., Graff G., Daines C.L., Dunitz J.M., Borowitz D., Orenstein D.M., Abdulhamid I., Noe J. (2018). Effects of an Antioxidant-enriched Multivitamin in Cystic Fibrosis. A Randomized, Controlled, Multicenter Clinical Trial. Am. J. Respir. Crit. Care Med..

[107] Yu Y., Sun X., Gu J., Yu C., Wen Y., Gao Y., Xia Q., Kong X. (2016). Deficiency of DJ-1 Ameliorates Liver Fibrosis through Inhibition of Hepatic ROS Production and Inflammation. Int. J. Biol. Sci..

[108] Kikuchi N., Ishii Y., Morishima Y., Yageta Y., Haraguchi N., Itoh K., Yamamoto M., Hizawa N. (2010). Nrf2 protects against pulmonary fibrosis by regulating the lung oxidant level and Th1/Th2 balance. Respir. Res..

[109] Liu L., Zhang X., Zhang R., Wang L., Zhi S., Feng X., Liu X., Shen Y., Hao J. (2023). Sohlh2 promotes pulmonary fibrosis via repression of p62/Keap1/Nrf2 mediated anti-oxidative signaling pathway. Cell Death Dis..

[110] Zhang C.Y., Zhong W.J., Liu Y.B., Duan J.X., Jiang N., Yang H.H., Ma S.C., Jin L., Hong J.R., Zhou Y. (2023). EETs alleviate alveolar epithelial cell senescence by inhibiting endoplasmic reticulum stress through the Trim25/Keap1/Nrf2 axis. Redox Biol..

[111] Liu X., Huang Y., Zhao X., Guan Y., Li Y., Yuan L., Wang C., Ma C., Ma E. (2024). Sodium cromoglycate exerts anti-pulmonary fibrosis effects by targeting the Keap1 protein to activate Nrf2 signaling. Bioorganic Chem..

[112] El-Horany H.E., Atef M.M., Abdel Ghafar M.T., Fouda M.H., Nasef N.A., Hegab I.I., Helal D.S., Elseady W., Hafez Y.M., Hagag R.Y. (2023). Empagliflozin Ameliorates Bleomycin-Induced Pulmonary Fibrosis in Rats by Modulating Sesn2/AMPK/Nrf2 Signaling and Targeting Ferroptosis and Autophagy. Int. J. Mol. Sci..

[113] Zhang X., Xiong D., Deng L., Qian R., Tang S., Liu W., Li Y., Liu L., Xie W., Lin M. (2025). Lysionotin attenuates bleomycin-induced pulmonary fibrosis by activating AMPK/Nrf2 pathway. Sci. Rep..

[114] He X., Wang L., Szklarz G., Bi Y., Ma Q. (2012). Resveratrol inhibits paraquat-induced oxidative stress and fibrogenic response by activating the nuclear factor erythroid 2-related factor 2 pathway. J. Pharmacol. Exp. Ther..

[115] Chien L.H., Deng J.S., Jiang W.P., Chou Y.N., Lin J.G., Huang G.J. (2023). Evaluation of lung protection of Sanghuangporus sanghuang through TLR4/NF-κB/MAPK, keap1/Nrf2/HO-1, CaMKK/AMPK/Sirt1, and TGF-β/SMAD3 signaling pathways mediating apoptosis and autophagy. Biomed. Pharmacother. Biomed. Pharmacother..

[116] Sriram N., Kalayarasan S., Sudhandiran G. (2009). Epigallocatechin-3-gallate augments antioxidant activities and inhibits inflammation during bleomycin-induced experimental pulmonary fibrosis through Nrf2-Keap1 signaling. Pulm. Pharmacol. Ther..

[117] Pandey J., Larson-Casey J.L., Patil M.H., Joshi R., Jiang C.S., Zhou Y., He C., Carter A.B. (2023). NOX4-TIM23 interaction regulates NOX4 mitochondrial import and metabolic reprogramming. J. Biol. Chem..

[118] Huang Y., Li Y., Lou A., Wang G.Z., Hu Y., Zhang Y., Huang W., Wang J., Li Y., Zhu X. (2020). Alamandine attenuates hepatic fibrosis by regulating autophagy induced by NOX4-dependent ROS. Clin. Sci..

[119] Agrawal A., Mabalirajan U. (2016). Rejuvenating cellular respiration for optimizing respiratory function: Targeting mitochondria. Am. J. Physiol. Lung Cell. Mol. Physiol..

[120] Afratis N.A., Parikh S., Adir I., Parikh R., Solomonov I., Kollet O., Gelb S., Sade Y., Vaknine H., Zemser-Werner V. (2025). Biselective remodeling of the melanoma tumor microenvironment prevents metastasis and enhances immune activation in mouse models. Sci. Transl. Med..

[121] Linder M., Pogge von Strandmann E. (2021). The Role of Extracellular HSP70 in the Function of Tumor-Associated Immune Cells. Cancers.

[122] Zhong B., Zhou J.Q., Lyu X., Liu H., Yuan K., Guo M.L., Duncan S.R., Sanders Y.Y. (2024). Anti-Heat Shock Protein 70 Autoantibodies from Patients with Idiopathic Pulmonary Fibrosis Epigenetically Enhance Lung Fibroblast Apoptosis Resistance and Bcl-2 Expression. J. Immunol..

[123] Monteleone G., Cameli P., Bonella F. (2025). The role of heat shock protein 90 in idiopathic pulmonary fibrosis: State of the art. Eur. Respir. Rev..

[124] Bellaye P.S., Shimbori C., Yanagihara T., Carlson D.A., Hughes P., Upagupta C., Sato S., Wheildon N., Haystead T., Ask K. (2018). Synergistic role of HSP90α and HSP90β to promote myofibroblast persistence in lung fibrosis. Eur. Respir. J..

[125] Lee J.Y., Reyes N.S., Ravishankar S., Zhou M., Krasilnikov M., Ringler C., Pohan G., Wilson C., Ang K.K., Wolters P.J. (2024). An in vivo screening platform identifies senolytic compounds that target *p16*^INK4a+^ fibroblasts in lung fibrosis. J. Clin. Investig..

[126] Lin J.H., Liu C.C., Liu C.Y., Hsu T.W., Yeh Y.C., How C.K., Hsu H.S., Hung S.C. (2024). Selenite selectively kills lung fibroblasts to treat bleomycin-induced pulmonary fibrosis. Redox Biol..

[127] Xu M., Zhang D., Yan J. (2024). Targeting ferroptosis using Chinese herbal compounds to treat respiratory diseases. Phytomedicine Int. J. Phytother. Phytopharm..

[128] Ciofu O., Smith S., Lykkesfeldt J. (2019). Antioxidant supplementation for lung disease in cystic fibrosis. Cochrane Database Syst. Rev..

[129] Lu S.C. (2009). Regulation of glutathione synthesis. Mol. Asp. Med..

[130] Calverley P., Rogliani P., Papi A. (2021). Safety of N-Acetylcysteine at High Doses in Chronic Respiratory Diseases: A Review. Drug Saf..

[131] Oldham J.M., Ma S.F., Martinez F.J., Anstrom K.J., Raghu G., Schwartz D.A., Valenzi E., Witt L., Lee C., Vij R. (2015). TOLLIP, MUC5B, and the Response to N-Acetylcysteine among Individuals with Idiopathic Pulmonary Fibrosis. Am. J. Respir. Crit. Care Med..

[132] Tang L., Zhang D., Zhang Y., Peng Y., Li M., Song H., Chen H., Li W., Li X. (2023). Vitamin D3 alleviates lung fibrosis of type 2 diabetic rats via SIRT3 mediated suppression of pyroptosis. Apoptosis Int. J. Program. Cell Death.

[133] Davies A.M., Holt A.G. (2018). Why antioxidant therapies have failed in clinical trials. J. Theor. Biol..

[134] Zhong T., Zhang W., Guo H., Pan X., Chen X., He Q., Yang B., Ding L. (2022). The regulatory and modulatory roles of TRP family channels in malignant tumors and relevant therapeutic strategies. Acta Pharm. Sin. B.

[135] Leon-Icaza S.A., Frétaud M., Cornélie S., Bureau C., Yatime L., Floto R.A., Renshaw S.A., Herrmann J.L., Langevin C., Cougoule C. (2025). Curcumin-mediated NRF2 induction limits inflammatory damage in, preclinical models of cystic fibrosis. Biomed. Pharmacother. Biomed. Pharmacother..

[136] Ma Y., Luo L., Liu X., Li H., Zeng Z., He X., Zhan Z., Chen Y. (2021). Pirfenidone mediates cigarette smoke extract induced inflammation and oxidative stress in vitro and in vivo. Int. Immunopharmacol..

[137] Veith C., Drent M., Bast A., van Schooten F.J., Boots A.W. (2017). The disturbed redox-balance in pulmonary fibrosis is modulated by the plant flavonoid quercetin. Toxicol. Appl. Pharmacol..

[138] Hao Y., Song K., Tan X., Ren L., Guo X., Zhou C., Li H., Wen J., Meng Y., Lin M. (2022). Reactive Oxygen Species-Responsive Polypeptide Drug Delivery System Targeted Activated Hepatic Stellate Cells to Ameliorate Liver Fibrosis. ACS Nano.

[139] Qi L., Duan B.W., Wang H., Liu Y.J., Han H., Han M.M., Xing L., Jiang H.L., Pandol S.J., Li L. (2024). Reactive Oxygen Species-Responsive Nanoparticles Toward Extracellular Matrix Normalization for Pancreatic Fibrosis Regression. Adv. Sci..

[140] Fang Y.F., Zhang C., Han M.M., Wang Y., Zhou T.J., Xing L., Wei N., Wang J., Jeong J.H., Zhou F. (2025). Engineered MSCs Break Endothelial-Myofibroblast Crosstalk in Pulmonary Fibrosis: Reconstructing the Vascular Niche. Adv. Mater..

[141] Hou J., Cong Y., Ji J., Liu Y., Hong H., Han X. (2024). Spatial targeting of fibrosis-promoting macrophages with nanoscale metal-organic frameworks for idiopathic pulmonary fibrosis therapy. Acta Biomater..

[142] Xu Y., Chen J., Jiang W., Zhao Y., Yang C., Wu Y., Li Q., Zhu C. (2022). Multiplexing Nanodrug Ameliorates Liver Fibrosis via ROS Elimination and Inflammation Suppression. Small.

[143] Vats S., Galli T. (2021). Introducing secretory reticulophagy/ER-phagy (SERP), a VAMP7-dependent pathway involved in neurite growth. Autophagy.

[144] Guo L., Chen Y., Feng X., Sun D., Sun J., Mou S., Zhao K., An R. (2022). Oxidative stress-induced endothelial cells-derived exosomes accelerate skin flap survival through Lnc NEAT1-mediated promotion of endothelial progenitor cell function. Stem Cell Res. Ther..

